# Integrated Computational Study of the Light-Activated Structure of the AppA BLUF Domain and Its Spectral Signatures

**DOI:** 10.1021/acs.jpca.3c02385

**Published:** 2023-06-06

**Authors:** Shaima Hashem, Giovanni Battista Alteri, Lorenzo Cupellini, Benedetta Mennucci

**Affiliations:** Dipartimento di Chimica e Chimica Industriale, Universitá di Pisa, Via G. Moruzzi 13, 56124 Pisa, Italy

## Abstract

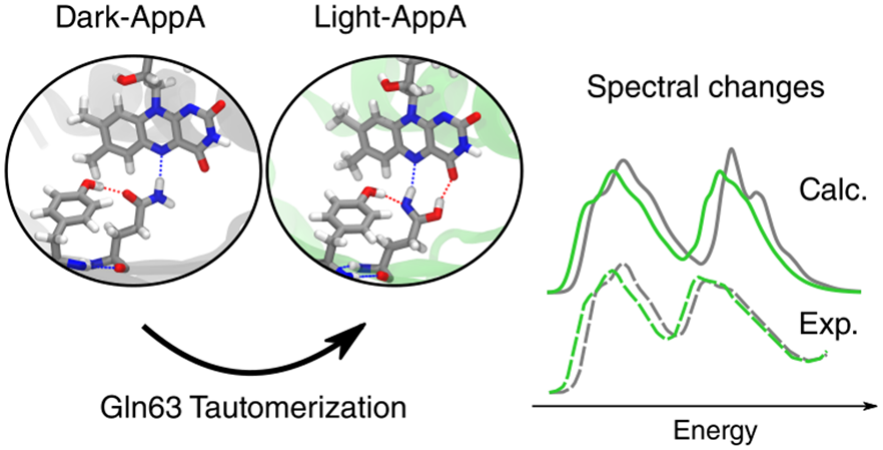

We apply an integrated approach combining microsecond MD simulations and (polarizable) QM/MM calculations of NMR, FTIR, and UV–vis spectra to validate the structure of the light-activated form of the AppA photoreceptor, an example of blue light using flavin (BLUF) protein domain. The latter photoactivate through a proton-coupled electron transfer (PCET) that results in a tautomerization of a conserved glutamine residue in the active site, but this mechanism has never been spectroscopically proven for AppA, which has been always considered as an exception. Our simulations instead confirm that the spectral features observed upon AppA photoactivation are indeed directly connected to the tautomer form of glutamine as predicted by the PCET mechanism. In addition, we observe small but significant changes in the AppA structure, which are transmitted from the flavin binding pocket to the surface of the protein.

## 1 Introduction

The accurate simulation of spectroscopies of systems of increasing complexity is now a reality, especially because of the development of efficient hybrid methods that combine the accuracy of quantum mechanical descriptions with the computational feasibility of molecular mechanics (MM) force fields.^1^ Since their very first applications, QM/MM methods have shown to be particularly suited to describe properties and processes of biological systems.^2−8^ However, the accurate simulation of the effects of the biological matrix (and the surrounding solvent) on the specific spectroscopic property of the embedded molecule(s) is not sufficient to achieve a complete picture of the spectral signatures of the system. A second aspect that has always to be considered in the simulation is the dynamic nature of the system and the effects that temperature-dependent fluctuations have on the final spectra. A very effective way to achieve such a description is to integrate QM/MM calculations with long molecular dynamics simulations of the whole system.

Here we show that this integrated method can indeed represent a powerful tool to investigate biological processes involving uncertain or even unknown structures. In particular, we integrate microsecond MD simulations and (polarizable) QM/MM calculations of NMR, FTIR, and UV–vis spectra to validate the structure of the light-activated form of the AppA photoreceptor. AppA is a protein that controls photosynthesis gene expression in purple bacteria, and it contains one of the most studied blue light using flavin (BLUF) domains. Contrary to other photoreceptors using flavin as chromophore,^9−12^ in the dark- and light-adapted structures of BLUF domains, including AppA, flavin does not display any structural or chemical change, but its spectroscopic responses are changed.^13^ Namely, by moving from the light to the dark state, a red-shift of almost 15 nm is observed in the flavin absorption spectrum and a 20 cm^–1^ red-shift in the infrared (IR) frequency corresponding to its carbonyl stretching mode.^14−16^ The two pieces of evidence suggest a change in the protein pocket binding the chromophore, whereas the flavin remains in its oxidized state. However, the real photoactivation mechanism and the light-adapted structure of AppA are still not known. As a matter of fact, the investigation of AppA photoactivation has proven difficult more than for other BLUF proteins due to two enigmas.

The first enigma is about the structure of the dark-adapted state. Two crystallographic structures for the BLUF domain of AppA (AppA-BLUF) have been resolved,^17,18^ which differ in the residues forming the flavin binding pocket. In both cases, the active site is composed of the flavin cofactor, either as mononucleotide (FMN) or flavin adenine dinucleotide (FAD), plus a tyrosine (Tyr21) and a glutamine (Gln63). However, in one of the structures^17^ a tryptophan residue (Trp104) is also at a close distance from the flavin, while in the second structure^18^ Trp104 is replaced with a methionine residue (Met106). Another difference between the two structures is the orientation of the Gln63 residue. In the structure including tryptophan, the glutamine-NH2 group faces Tyr21,^17^ while in the other, Gln63 is rotated by 180°, creating a hydrogen bond between Tyr21 and the carbonyl group of glutamine.^18^

The second enigma concerns the photoactivation mechanism. For several BLUF domains, both experiments and computational studies strongly suggest that a proton-coupled electron transfer (PCET) process occurs at the excited state, finally leading to a keto–enol tautomerization of Gln63.^11,19−26^ However, this PCET process has never been experimentally proven for AppA-BLUF, which led several authors to hypothesize alternative mechanisms for this protein.^27−30^ Some authors proposed that only a rotation of the Gln63 occurs in the light-adapted state,^17,31,32^ whereas others proposed a Gln tautomerization, accompanied by side-chain rotation.^15,29,33^

**Figure 1:**
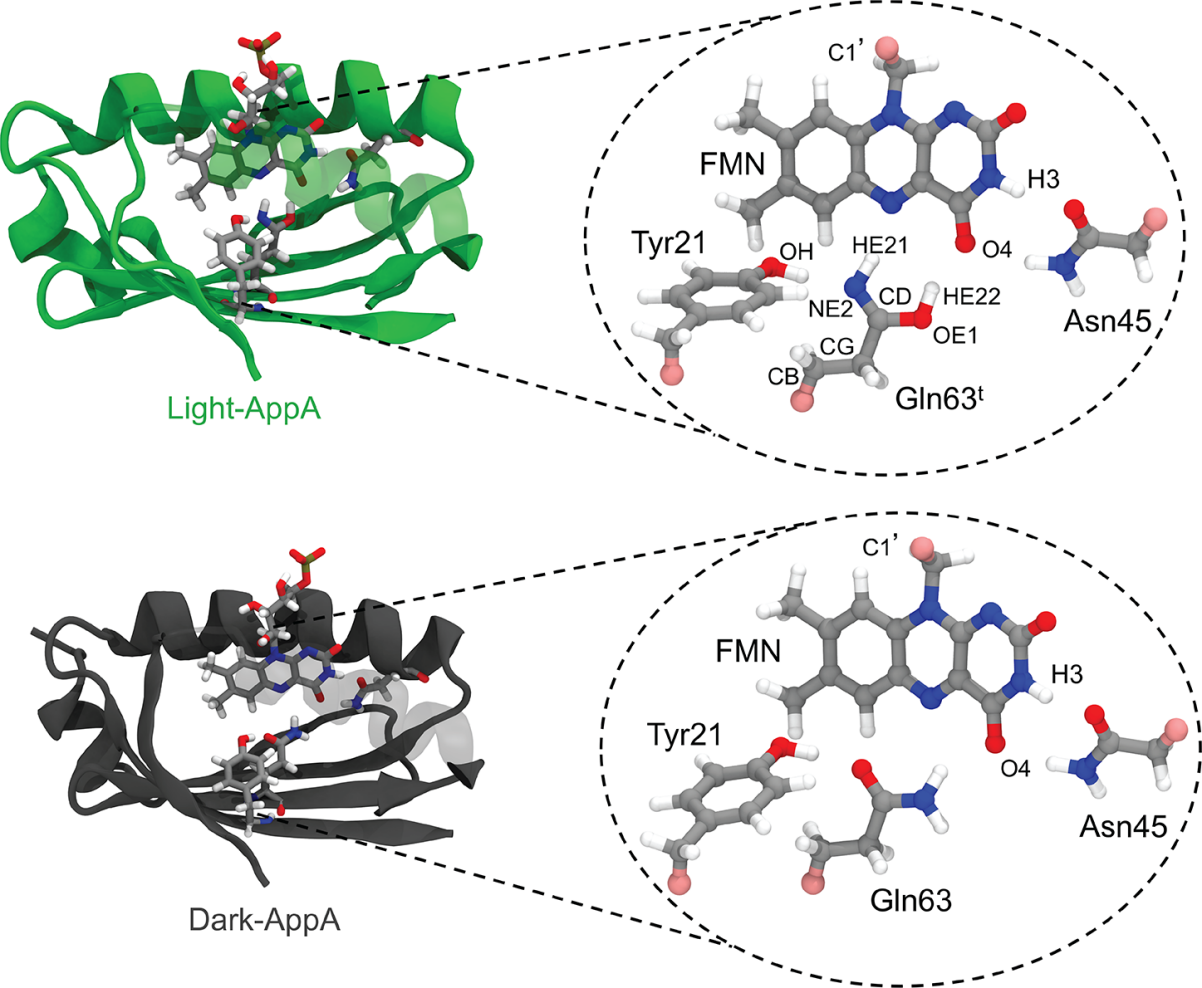
Structure of AppA-BLUF in the light-induced state light-AppA (top) and in the dark state dark-AppA (bottom) containing the flavin chromophore and the main interacting residues in the active site (Tyr21, Gln63, and Asn45). The insets show the QM subsystem used in the optimization and the excitation calculations. Protein atoms are labeled according to the standard PDB atom names. The two structures were extracted from the MD simulations of light-AppA (this work) and of dark-AppA (ref (34)).

To resolve the structural ambiguity of the dark state, we have recently investigated the two proposed structures using molecular dynamics (MD) simulations and NMR, IR, and UV–vis spectroscopic calculations. Our simulations confirmed that the dark-adapted state of AppA is compatible with the structure by Jung et al.,^18^ containing Met106 in the binding site instead of the Trp104.^34^ Very recently, starting from this dark-adapted state, we have simulated the photoactivation mechanism using polarizable QM/MM dynamics simulations in the excited and ground states.^35^ These simulations showed that the PCET mechanism is indeed possible for the AppA protein, suggesting a conserved mechanism among different BLUF domains. This mechanism implies that Gln63 can undergo tautomerization only if the side chain rotates as well. The proposed active site in the light-induced state of AppA-BLUF (from now on, light-AppA) is shown in Figure 1 and compared to the dark-adapted state (dark-AppA). Nevertheless, it remains to be proven whether the proposed structure for the light-induced state can explain the spectroscopic differences observed upon photoactivation. Additionally, it is still unclear how a small, rather local change such as tautomerization of Gln63 can propagate to the entire protein and determine photoactivation.

To confirm the proposed PCET mechanism and investigate its consequences on the structure of the protein, here we perform MD simulations of the proposed light-induced state, and we compare its NMR, IR, and UV–vis signals with those calculated for the dark state to have a direct comparison with the measured spectroscopic features used to characterize the dark-to-light change. Our calculations reproduce all the main spectroscopic fingerprints of the photoactivated state, including the red-shift observed in UV–vis and IR spectra, and trace them back to the change in the hydrogen bond pattern experienced upon tautomerization of Gln63. Furthermore, the MD simulations suggest that the change in the BLUF domain upon photoactivation is rather local and mainly induced by small differences in the binding mode of flavin in the active site.

## 2 Methods

### 2.1 MD Simulations of Light-AppA

The structure of light-AppA, containing Gln63 in its imidic acid tautomer form (Gln63^*t*^), was obtained by modeling and parametrizing a new force field for Gln63^*t*^ and by keeping the rest of the protein unchanged from the dark state. The protocol of the simulations of the dark-adapted state is reported in our previous study.^34^ For light-AppA, a similar protocol was applied. The only difference is that a distance restraint between Tyr21 HH and Gln63^*t*^ NE2 and a torsional restraint on the Gln63^*t*^ CB–CG–CD–OE1 dihedral were introduced during the production, with force constants of 10 kcal/(mol Å^2^) and 100 kcal/(mol rad^2^), respectively. This was found necessary to enforce the direction and strength of the H-bond between Gln63^*t*^ and Tyr21, which were verified *a posteriori* by inspecting QM/MM optimized structures (see below). Three replicas were performed for light-AppA for a total simulation time (production only) of 7.5 μs (3 × 2.5 μs).

Parametrization of the charge, bond, and angle parameters for the glutamine imidic acid tautomer was performed in order to ensure compatibility with ff14SB. The bonded parameters involving the imidic group were taken from GAFF.^36^ The charges were fitted following the protocol used in Amber protein force fields.^37^ Two conformers of the tautomer were generated, and the charges were fitted using the RESP procedure based on the electrostatic potential calculated at the B3LYP/6-311G(d,p) level of theory. All the parameters of the glutamine imidic acid tautomer are reported in the Supporting Information.

Hydrogen-bond (HB) networks were analyzed to identify the changes that are induced by the formation of the light-excited state. The frequency of occurrence (or occupancy) of HB interactions was calculated for the residue pairs that are interacting and surrounding the flavin chromophore, using a cutoff of 135° on the hydrogen–acceptor–donor angle and of 3.2 Å on the donor–acceptor distance. To depict the change occurring in the angle of the two α-helices between the dark and the light states, a vector for each helix was defined for each state. Each vector was described by four consecutive C_α_ atoms at the beginning and at the end of each helix. The selected C_α_ atoms on the two helices are Asn45, Ala46, Arg47, Ala48, Leu31, Arg32, Asp33, Leu34, Arg68, Pro69, Ala70, Ala71, Arg81, Asp82, Arg83, and Arg84. Structural changes involving the β-sheet were measured by the dihedral angle between four C_α_ atoms on the β-sheet, Ser18, Leu50, Gly59, and Glu89 (Figure 5a), and the distribution of this angle between the dark and the light simulations is reported in Figure 5b.

### 2.2 Simulation of Spectroscopies

#### Optimizations and Vibrational Frequency Calculations

Geometry optimizations were performed using a ONIOM(QM:MM) scheme^38^ on 200 configurations extracted from the last 500 ns of the three MD replica simulations of light-AppA. In these optimizations, the QM subsystem comprising the isoalloxazine ring, including the C1′ atom and the side chains of the Gln63^*t*^, Tyr21, and Asn45 residues within the binding pocket, was treated at the B3LYP/6-31G(d)-D3 level of theory^39^ (see Figure 1). The rest of the protein and the solvent within 30 Å, excluding the Na^+^ and Cl^–^ ions, were incorporated in the MM part and kept frozen. The flavin mononucleotide (FMN) ribityl tail was also treated at MM level but allowed to move. The MM part was described with the same force field^40,41^ as in the MD simulations. Subsequently, harmonic frequencies were computed starting from the optimized configurations, at the same level of theory. Identical calculations were performed for 100 configurations extracted from MD simulations of the dark-AppA of a previous study.^34^ All calculations were performed with Gaussian 16.^42^

#### NMR Calculations

The 200 optimized configurations were used to compute the chemical shifts for the protons of the FMN ring and the proton of Tyr21 side chain using a polarizable QM/MM model. The QM part consisted of the isoalloxazine ring and the side chains of Gln63/Gln63^*t*^, Tyr21, and Asn45 residues and was treated at the B3LYP/6-311+G(d,p) level. The latter has been shown to properly reproduce MP2/6-311G(d,p) calculations.^43^ The protein, ions, and water molecules within 40 Å of the chromophore were treated using the same polarizable MM model used in a previous study on dark-AppA.^43^ All calculations were performed with a locally modified version of Gaussian 16 in which the polarizable QM/MM has been implemented.^44,45^

#### Excited-State Calculations

Excited-state calculations were performed on the same structures used for the NMR calculations but changing the QM level into ωB97X-D/6-31+G(d) and using AMOEBA as a polarizable force field.^46^ Additional calculations at the ADC(2) and CC2 levels with different basis sets were performed using a nonpolarizable QM/MM description (see the Supporting Information Section S2). The simulation of the spectra was obtained through the same approach used in the previous study of dark-AppA.^34^ Namely, the vibronic couplings with all the normal modes were obtained through the spectral density, calculated using a vertical gradient (VG) approximation^47^ and the ONIOM(QM:MM) scheme described above. The absorption spectra were finally computed by convoluting the resulting homogeneous line shape with the inhomogeneous distribution of vertical excitation energies computed along the MD trajectories. All calculations were performed with a locally modified version of Gaussian 16, except for CC2 and ADC(2) calculations which were performed with the ricc2 module^48,49^ of TURBOMOLE.^50,51^

## 3 Results and Discussion

### 3.1 Spectroscopic Signatures of the Light State

In a recent study, we have shown that a PCET mechanism of photoactivation is possible for AppA.^35^ As shown in Figure 1, this mechanism results in an important change in the glutamine placed very close to the flavin, which transforms in its ZZ imidic acid tautomer. To explore the conformational landscape of this photoproduct (light-AppA) and compare it to the dark-adapted state (dark-AppA), we performed three classical MD simulations (replicas) for a total of 7.5 μs of simulation (see the Methods section). The resulting sampling was used to assess the validity of our model for light-AppA by simulating the different spectroscopic signatures that characterize the light-induced state. We used 200 configurations obtained from the last 500 ns of three MD light simulations to perform geometry optimizations and subsequent spectroscopy calculations (see the Methods section).

#### NMR Chemical Shifts and IR Frequencies

As observed previously,^43^ including the closest H-bonding residues in the QM part is paramount to describing correctly the chemical shifts of flavin in AppA. For this reason, as stated in the Methods section, we included Gln63, Asn45, and Tyr21 in the QM part in all polarizable QM/MM calculations. The results, averaged along the MDs, are reported in Table 1 together with those obtained for dark-AppA.^43^

**Table 1:** Experimental and Calculated NMR Chemical Shifts and IR Frequencies Dark-Appa and Light-AppA^a^

		NMR		IR
		FMN H_3_ (ppm)	Tyr HH (ppm)	C_4_=O_4_ (cm^–1^)
exptl	dark	11.16		1707^b^/1709^c^
	light	11.8	10.22	1684^b^/1695^c^
	diff	0.64		–23^b^/–14^c^
calcd	dark	11.6 ± 0.2	7.7 ± 0.2	1753 ± 3
	light	11.9 ± 0.1	11.1 ± 0.2	1727 ± 2
	diff	0.3 ± 0.2	3.5 ± 0.2	–26 ± 3

a For calculated data, we report the average on MD frames and the bootstrapped 95% confidence intervals. Experimental values of IR frequencies are taken from ref (32), and chemical shifts are taken from refs (31 and 52). Chemical shifts are reported with respect to TMS.

b Flavin frequencies reconstructed from deconvolution in ref (32).

c Raw negative/positive peaks in ref (32).

A ∼0.6 ppm increase in the chemical shift of flavin H_3_ has been observed in NMR experiments as the only relevant change in flavin upon photoactivation.^31^ This increase is reproduced by our calculations (Table 1), although it is smaller than in the experiment, even considering the confidence interval for this difference, which is between 0.1 and 0.5 ppm. We note, however, that the uncertainty is likely underestimated as it does not account for correlations among the data. The multimodal distribution of chemical shifts (Figure S5) suggests that several slowly exchanging conformations are present, and the uncertainty in their populations impacts the chemical shift.

The increase in the H_3_ chemical shift is accompanied by a slight shortening of the H-bond with Asn45 (Figure S2a). As predicted previously on the basis of the dark structure only,^43^ the H_3_ chemical shift increases with shortening H-bond to Asn45. However, we also observed an upshift of H_3_ in the ligth-AppA structures that have a similar H-bond length to dark-AppA. Therefore, the chemical shift change on H_3_ is also related to a stronger hydrogen bond between O_4_ and the Gln63^*t*^ side chain (Figure S1), which results in the modification of the electronic structure of the flavin isoalloxazine ring.

In addition to flavin H_3_, we consider the hydroxyl proton of Tyr21, which is experimentally observed only in the light state^52^ at 10.22 ppm. Our calculations also predict a strongly deshielded proton (Table 1), in contrast to dark-AppA, which presents a much lower chemical shift. Although calculations overshoot the value of this chemical shift by almost 1 ppm, they indicate that the Tyr21 hydroxyl group is involved in a strong hydrogen bond. Indeed, the H-bond between Tyr21-OH and Gln63^*t*^ is tighter in light-AppA, with a slightly shorter distance within the optimized structures (Figure S2b). This observation is in agreement with the calculations of Iwata et al.^15^ Another reason for the increased chemical shift on the Tyr21 hydroxyl proton is the change of the functional group that is H-bonded to it. The H-bond to the unusual imidic group of Gln63^*t*^ affects the electronic density of the OH group differently from the carboxyl of the amide group found in dark-AppA. Taken together, these chemical shifts suggest that the H-bond rearrangement following tautomerization of Gln63 is responsible for the chemical shift variations observed in light-AppA.

The dark-to-light conversion is also characterized by a typical IR red-shift of ∼20 cm^–1^ of the carbonyl C_4_=O_4_ stretching mode, measured through light-induced FTIR difference spectra.^14,15,32,53^ This signal was attributed directly to the stronger hydrogen bond between Gln63^*t*^ and the flavin C_4_=O_4_.^14,54^ Our calculations confirm this picture: in light-AppA, an additional hydrogen bond is formed between Gln63^*t*^ HE22 and FMN O_4_. To confirm the link between H-bonding and stretching frequency, we computed the harmonic frequencies for the C_4_=O_4_ stretching in dark-AppA and light-AppA (Table 1). Our calculations predict a downshift by ∼26 cm^–1^, which substantially agrees with the red-shift observed passing from dark- to light-AppA in the experiments.

#### Electronic Absorption

We then focused on the electronic absorption spectrum for dark- and light-AppA. The electronic absorption of oxidized flavin in BLUF domains features two distinct bands in the visible/near-UV range at ∼450 nm (2.8 eV) and ∼360 nm (3.4 eV).^55,56^ Both bands experience a red-shift upon photoconversion to the light state (see Table 2), although the redmost band shows the largest red-shift (10–15 nm).^14,55,56^

**Table 2:** Experimental^55^ and Calculated TD-ωB97XD/6-31+G(d)/AMOEBA Vertical Excitation Energies (eV) of the First Two Bright States (S_1_ and S_2′_) in Dark-Appa and Light-AppA^a^

Δ*E* (eV)	system	S_1_	S_2′_	L–D (S_1_)	L–D (S_2′_)
exptl	dark	2.79	3.40		
	light	2.71	3.35	–0.08	–0.05
calcd	dark	3.20 ± 0.007	3.95 ± 0.01		
	light	3.13 ± 0.004	3.90 ± 0.01	–0.07	–0.05
calcd	dark	3.27 ± 0.005	4.11 ± 0.01		
(w/o Gln63)	light	3.27 ± 0.005	4.09 ± 0.01	0.00	–0.02

a Calculated excitation energies refer to an average over the optimized MD structures, and the corresponding bootstrapped 95% confidence intervals are reported. The last two rows are the results obtained by completely removing Gln63/Gln63^*t*^ from the calculations of dark/light structures.

Before simulating the spectra in the dark and light states, we first analyzed the nature of the excited states of FMN using dark-AppA. Preliminary calculations (Table S1) performed at the ωB97XD/6-31+G(d) level on the dark-AppA crystal structure showed that the first excited state (S_1_) is a bright state corresponding to the first absorption band of the measured spectrum, whereas the next bright state which corresponds to the second observed band is S_3_. At this level of theory, the excitation energies of the two bands are significantly overestimated, as observed before,^57^ particularly for the second band. This also leads to overestimating the energy difference between the first two bands. When using structures extracted from the MD trajectory, it is necessary to employ a more careful approach to assign excited states, as the oscillator strength of the second bright state is concentrated in either S_3_ or S_4_ depending on the selected structure. In the following, we refer to the second bright state as S_2′_ to avoid any confusion with the actual S_2_ state, which is always dark. For each structure, we identify S_2′_ with the state having the largest oscillator strength.

We first analyzed the effect of the level of theory on the computed excitation energies; we compared TD-ωB97XD excitation energies with TD-B3LYP and ADC(2) and CC2 results using a subset of the MD optimized structures. We also investigated the effect of the basis set. The QM/AMOEBA approach is not available for ADC(2) and CC2; all these calculations were therefore performed with an electrostatic embedding QM/MM, and only the flavin ring was included in the QM part. These results (Table S2) show that both CC2 and ADC(2) predict excitation energies close to the experiment for S_1_, but they systematically overshoot the S_2′_ state, resulting in a S_2′_–S_1_ difference of ∼1 eV, much larger than the experiment (∼0.6 eV). The overestimation of S_2′_–S_1_ difference is also found at the TD-DFT level, but it is smaller than with the other two methods. Between the TD-DFT methods, B3LYP shows the best agreement with experiments, but closer inspection showed that with this functional both bright states were strongly mixed with dark states. This issue was observed before,^57^ and it does not depend on the basis set—all that finally encouraged us to employ ωB97X-D/6-31+G(d) in subsequent calculations.

To compare the excitation energies of flavin in light-AppA and dark-AppA, we then performed QM/AMOEBA calculations including hydrogen-bonded residues Gln63 and Asn45 in the QM part, as well as Tyr21, in analogy with NMR calculations. We first analyze the average vertical excitation energy along the dark-AppA MD and the three light-AppA replicas (Table 2).

As expected from the previous analysis, the calculations overestimate the S_1_ and S_2′_ energies by ∼0.4 and ∼0.6 eV, respectively. However, looking at the difference between dark- and light-AppA, the calculated averages well reproduce the red-shift observed in the experiments^55^ for both absorption bands. To investigate the origin of the red-shift, we repeated the excited-state calculations by removing Gln63/Gln63^*t*^ from the dark/light-AppA structures. In these calculations (see Table 2) all the excitation energies are blue-shifted with respect to the full system, but by different amounts in the dark and light states. As a result, the shift in the first band disappears, and the one for the second band is reduced by more than half. This finding not only further confirms that the tautomerization of Gln63 is consistent with the light state of AppA, but it shows that the different H-bond pattern in tautomeric Gln is a direct cause of the observed absorption red-shift when moving from the dark to the light state.

**Figure 2:**
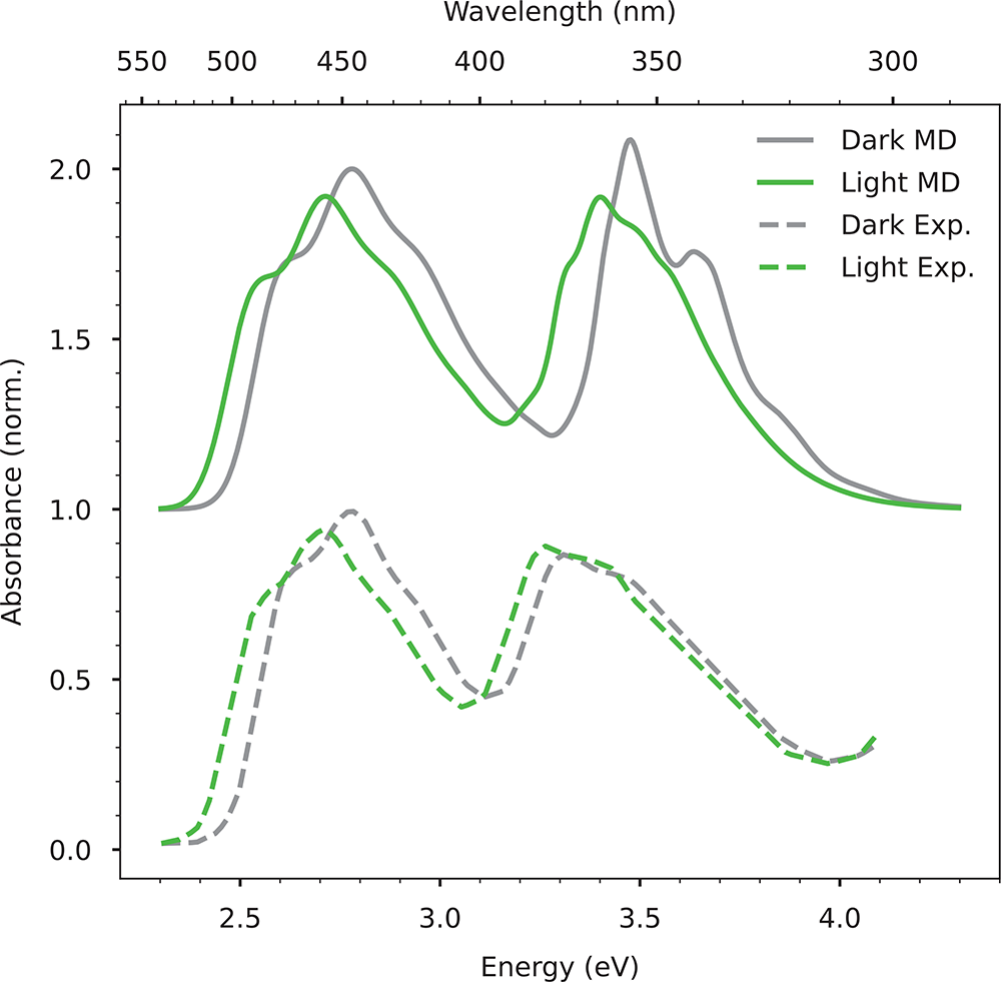
Comparison between the experimental (dashed lines) and the simulated absorption spectra (solid lines), for both dark (gray) and light (green) states. All calculated spectra were shifted in energy by −0.35 eV, to match the position of the first maximum in the dark state, and scaled by the same factor to match the intensity of the same band. The computed spectra are vertically offset by one unit for clarity.

As a final confirmation of our findings, we employed the same approach validated in a previous work for dark-AppA^34^ (see the Methods section) to simulate the absorption spectra of both dark and light states. The spectra were simulated including both S_1_ and S_2′_ energies and are compared to the experiments in Figure 2.

We first note that our calculations accurately reproduce the vibronic line shape of the first band, and the shift between maxima moving from dark- to light-AppA (∼0.08 eV) is identical to the experiment. Additionally, for the first band our calculations also reproduce the slight decrease in intensity observed in light-AppA. However, as expected from our results on vertical excitation energies, the second band is too blue-shifted relative to the first band, and the simulated line shapes show a worse agreement with the measured ones. Specifically, the calculated bands appear too narrow, especially for dark-AppA, which prevents a reproduction of the relative intensity of the two bands. A much better agreement is instead found for light-AppA. The less accurate reproduction of the line shape for the second band may be attributed to the difficulty in clearly identifying the corresponding bright state, as discussed above. Nevertheless, the shift between dark-AppA and light-Appa is reproduced also for the second band.

### 3.2 Protein Structural Changes upon Activation

The previous analysis shows that the obtained photoproduct indeed presents all the spectroscopic signatures that characterize the light-activated state of AppA. Starting from this finding, we proceed with the analysis of the structural changes that accompany the new state of the protein.

**Figure 3:**
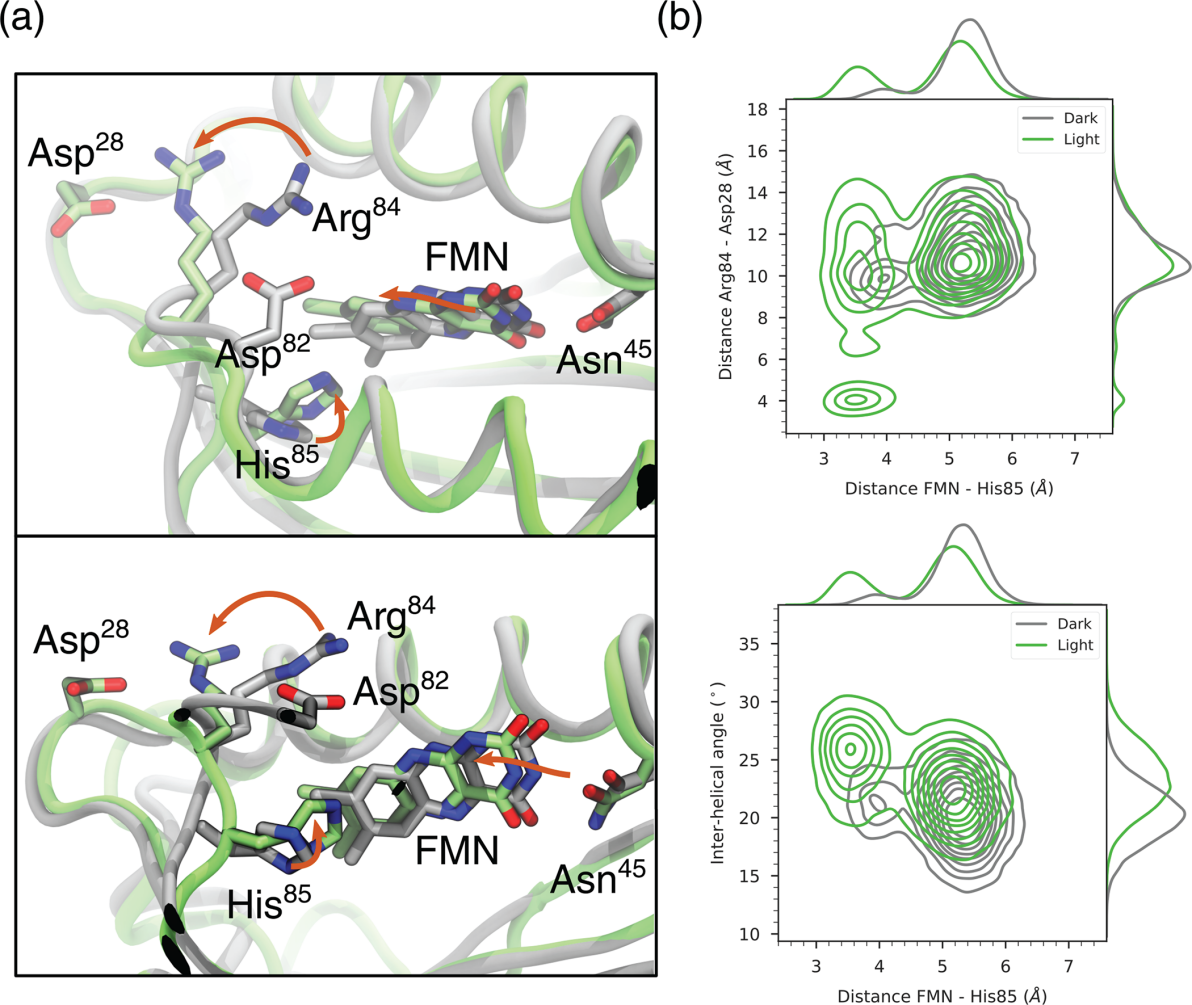
Dark–light differences. (a) Representative frames of the dark-state MD (gray) and of the tautomer Gln63 MD (green). Top/bottom panels show the top and side views, respectively. Orange arrows highlight the most important changes in passing from the dark to the light structure. (b) Distributions of relevant coordinates in the dark and light states, including all light-state MD. The FMN–His85 distance is computed between the benzene ring of FMN and the N_ε_ atom of His, the Arg–Asp distance is computed from the last carbon atom of each residue, and the interhelical angle is computed as the angle between two vectors following the two α-helices.

The three MD replicas suggest a more disordered dynamics for light-AppA, which can be visualized from the distribution of the RMSD calculated on the backbone atoms, excluding the flexible parts for each state in (Figure S3). This disordered dynamics can explore new conformations that are not seen in the dark state. One of these conformations is shown in Figure 3a compared to a representative structure of the dark state.

We can pinpoint several key movements characterizing this conformation. First, the flavin ring slides within the pocket toward His85, which establishes an interaction with the benzene ring of FMN. This conformation is only populated in the light-AppA MDs (Figure 3b), while it is only barely approached in dark-AppA. Visual inspection suggested that this conformation is formed as the result of a change in the hydrogen bond network around His85. Indeed, there is an intermittent loss of a number of hydrogen bonds during the light-AppA simulations, as depicted from their occurrences in Figure S4. Specifically, the breakage of the hydrogen bond between His85(NE2) and Ile79(O) allows the histidine to freely move and interact with the flavin. In addition, we can notice the movement of Arg84, which breaks its salt bridge with Asp82 and interacts with Asp28. As it clearly appears from Figure 3b (top), the Arg84–Asp28 salt bridge is only formed in light-AppA, and only when His85 interacts with flavin. Strikingly, a change in the environment of Asp82 was detected by time-resolved FTIR spectroscopy, and as such Asp82 was proposed as the site of signal transduction.^58^

**Figure 4:**
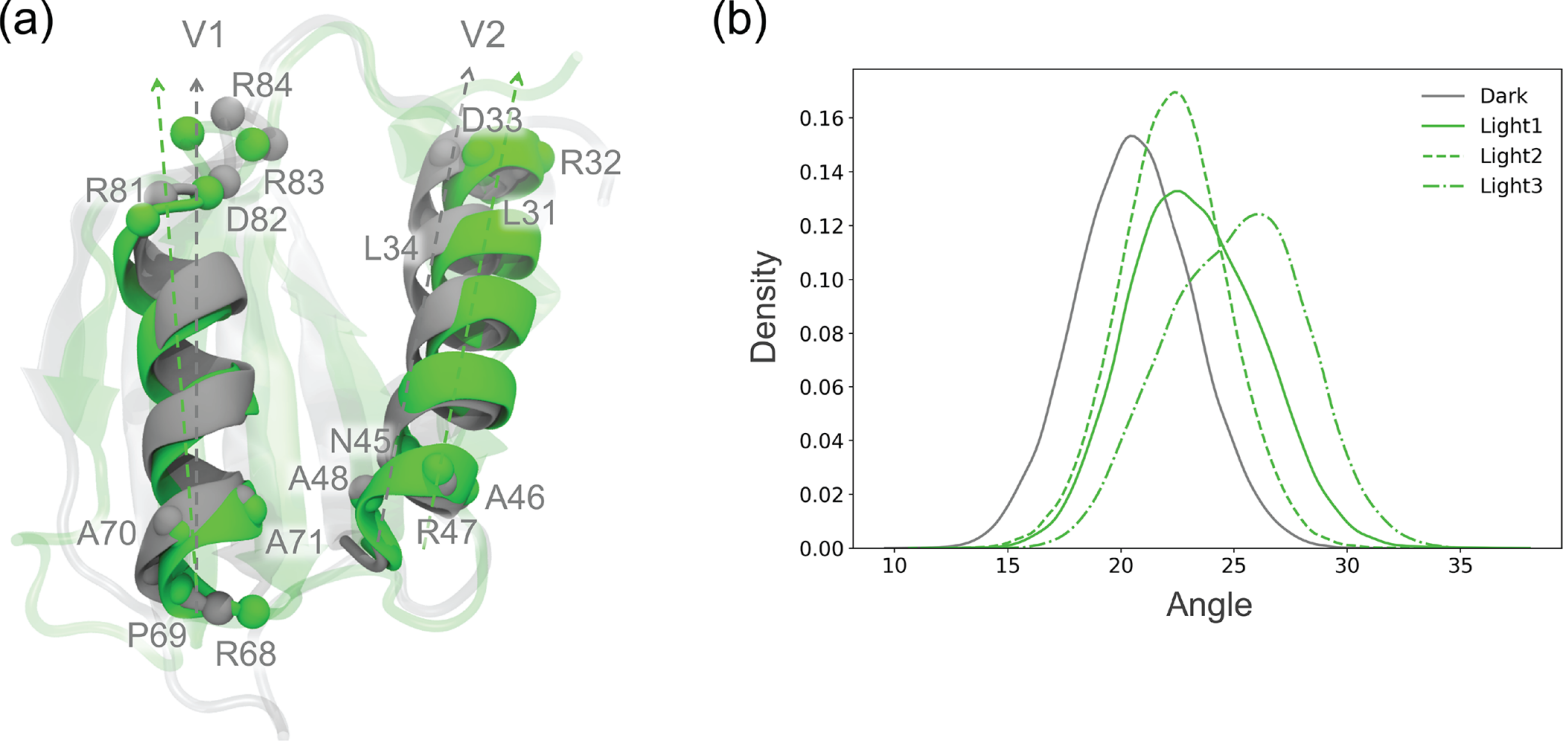
Structural changes in the α-helices. (a) Definition of the interhelical angle and representative structures from the dark-AppA and light-Appa dynamics. (b) Distribution of the interhelical angle for the dark state and for the three replicas of the light state.

The overall structure of the BLUF domain also changes. The angle formed by the two α-helices of the domain increases slightly in light-AppA (Figure 4), especially when the His85–FMN interaction is established. This suggests that the two helices can reorient to adapt to the movement of the flavin in its binding pocket. As a consequence, the helices can slightly open toward the C-terminal side and close at the opposite side.

**Figure 5:**
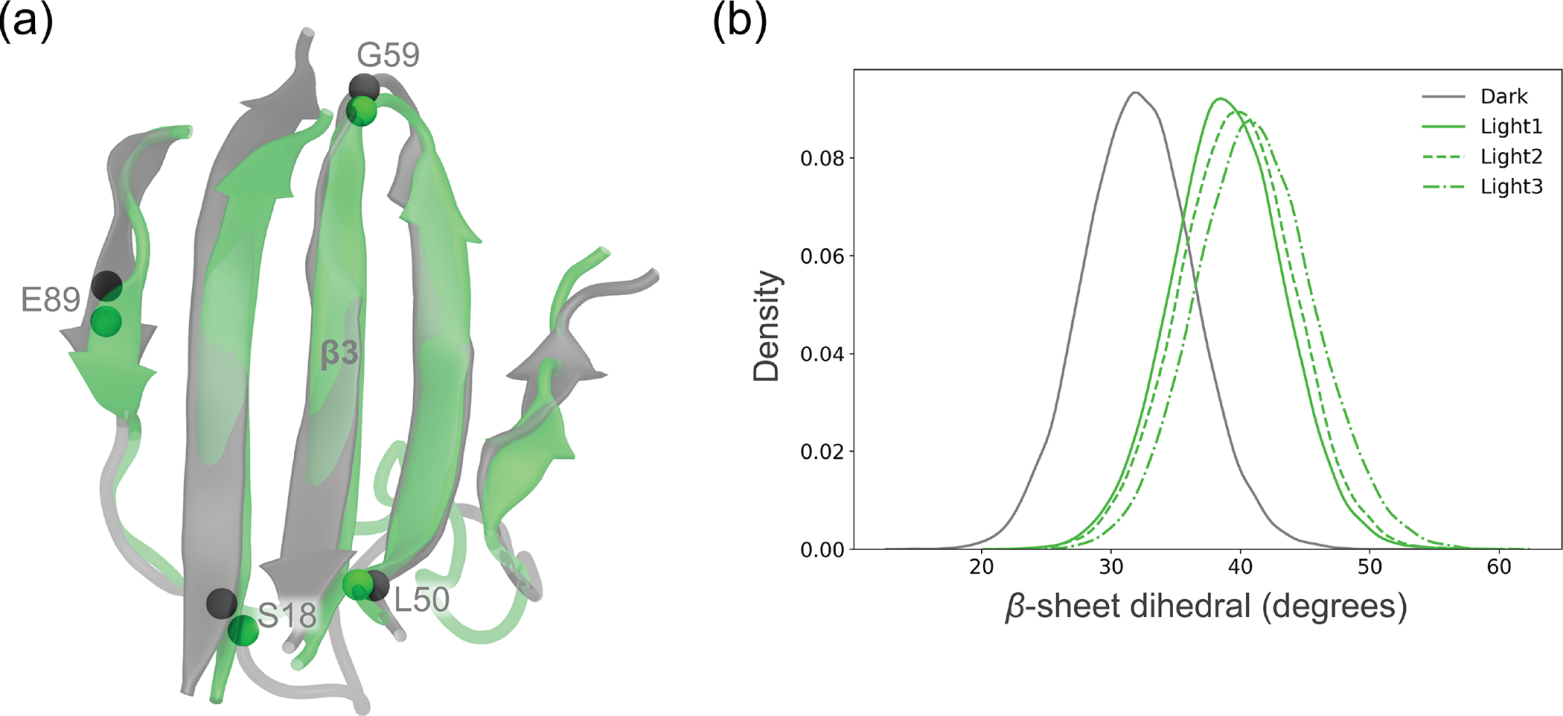
Structural changes in the β-sheet. (a) Definition of the dihedral angle of the β-sheet and representative structures from the dark-AppA and light AppA dynamics. (b) Distribution of the dihedral angle for the dark state and for the three replicas of the light state.

We observed an additional structural change located in the β-sheet, which appears more twisted in light-AppA simulations (Figure 5). We quantified this twisting by measuring the dihedral angle between four C_α_ atoms at the ends of the β-sheet (Figure 5a). The dihedral angle distribution is remarkably consistent between light-AppA MD replicas and significantly altered to larger values with respect to the dark-AppA simulation, meaning that the β-sheet is less planar in the light state. The twisting can be traced back to a weakening of the hydrogen-bond network between residues located along the β-strands. The occurrence of backbone hydrogen bonds in the residue pairs Leu24–Ser86 and Arg84–Ala26 (Figure S4) illustrates this rearrangement. Light-minus-dark FTIR difference spectra displayed changes in the protein backbone regions, which were assigned to the C=O modes of the β-sheet backbone.^32^ As Gln63 is located on the β3 strand of AppA-BLUF, the β-sheet signals were tentatively attributed to this strand.^54^ However, the significant changes in H-bond that we observed around Arg84 suggest that this residue may play a role in transmitting the active-site structural changes to the β-sheet.

Our simulations allow us to put forward a hierarchical model for AppA activation. First, the Gln63^*t*^ imidic acid tautomer binds flavin differently from the amide form, ultimately stabilizing a slightly different position for FMN in the binding pocket. This allows for the interaction with His85 and for the conformational change of Arg84, which then forms a new salt bridge with Asp28. The changes in the binding pocket are then transduced through Arg84 to the β-sheet, which experiences a distortion from planarity. Overall, the structural changes observed within our MD simulations are rather restricted. Clearly, the microsecond time scale simulated here might be too short to observe wider conformational rearrangements. Very small changes in the absorption spectrum of AppA-BLUF were observed at the millisecond time scale,^59^ i.e., 3 orders of magnitude longer than our current simulations. Nonetheless, our results agree with experimental evidence that AppA-BLUF alone shows only limited structural changes upon photoactivation.^60^

Finally, we note that our simulations only comprise the BLUF domain of AppA, which prevents us from investigating the activation mechanism beyond the domain level. Indeed, in AppA the BLUF domain is connected to the output C-terminal SCHIC domain executing the antirepressor function, and there is evidence of an interaction surface between these domains.^56,60^ In full-length AppA, dynamics in the 500 ms time scale were detected, which were not present in the BLUF domain,^59^ suggesting that the structural changes propagate to the C-terminal domain in this time frame. We speculate that the change of binding in Asp28 can modify the interactions at the interface with the C-terminal domain, resulting in a broader change.

## 4 Conclusions

By combining classical MD simulations and multiscale QM/MM simulations of IR, NMR, and UV–vis spectroscopic features, we have shown that all the main experimental pieces of evidence on the light-induced state of AppA can be properly reproduced by assuming the formation of a glutamine imidic acid tautomer at Gln63, as predicted by the PCET mechanism of photoactivation.^21,35^ Tautomerization of glutamine results in a modification of the hydrogen-bonding pattern around flavin, strengthening the Gln63–flavin interaction by the formation of two hydrogen bonds. Comparison of H-bond strength and observed chemical shifts also reveals a tightening of the Gln63–Tyr21 H-bond upon Gln63 tautomerization. The newly formed interactions on the O_4_ and N_5_ atoms also influence the electronic structure of the flavin, causing a red-shift of the two lowest absorption bands and an increase in the H_3_ chemical shift. Our results suggest that the tautomeric state of Gln63 directly influences the electronic structure of the flavin.

The MD simulations reveal small but significant changes in the structure of AppA-BLUF upon photoactivation. The altered H-bond pattern causes the flavin to move in a slightly different position within the binding pocket. In turn, this induces a change in the conformation of Arg84, located in a different region of the binding pocket, which reflects on its interaction with farther residues. Clearly, the fact that our simulations only include the BLUF domain of AppA prevents us from investigating how this propagation moves in the full-length protein. Further investigations accounting for the interaction of the BLUF domain with the rest of the protein are needed to achieve a complete picture of the cascade of structural changes finally leading to the activation of the biological function.

Our investigation shows that a careful integration of MD simulations with hybrid QM/MM calculations represents a powerful tool for investigating protein active sites in their metastable state, e.g., as obtained by photoactivation. A crucial aspect of this strategy is the connection of independent spectroscopic signatures into a consistent global picture, which on the one hand enhances the robustness of the observations and on the other hand allows a deeper understanding of the structure–spectroscopy relationships. We believe that such a strategy can be successfully employed for other photoreceptor proteins with short-lived light-activated states.

## Supporting Information Available

Additional details on MD simulations; additional details on QM/MM excited-state calculations; excitation energies and oscillator strengths for the first five singlets of FMN in dark-AppA calculated on the crystal structure; hydrogen-bond interactions in the active site; distribution of backbone RMSD; occurrence of selected hydrogen bonds; comparison between the vertical excitation energies for the bright states of FMN in dark-AppA computed at QM/MM using different QM levels of theory and different basis sets (PDF)
AMBER library and force-field modification files for the Gln tautomer (ZIP)
