# Supplementary material for: Integrated Computational Study of the Light-Activated Structure of the AppA BLUF Domain and Its Spectral Signatures
Source: J Phys Chem A. 2023 Jun 6;127(23):5065–74. doi: 10.1021/acs.jpca.3c02385 (PMC10278134; doi:10.1021/acs.jpca.3c02385)
Supplement: Supplementary file 1 — jp3c02385_si_001.pdf [file jp3c02385_si_001.pdf]

Supporting Information: Integrated Computational Study of the Light-activated Structure of the AppA BLUF Domain and its Spectral Signatures

Shaima Hashem, Giovanni Battista Alteri, Lorenzo Cupellini, and Benedetta Mennucci*

Dipartimento di Chimica e Chimica Industriale, Università di Pisa, Via G. Moruzzi 13, 56124 Pisa, Italy

E-mail: benedetta.mennucci@unipi.it

S1 Additional details on MD simulations

Preparation. All MD simulations were performed using Amber18[1,2]. The initial structure was extracted from our previous simulation of dark-AppA,[3] and only Gln63 was reprotonated as a glutamine tautomer (Gln^t). The dark-AppA structure was already solvated in an truncated octahedron box with TIP3P water molecules and NaCl counterions; the equilibrated solvent/ions distribution was kept during the preparation of light-AppA. The Amber ff14SB force field[4] was used to describe the protein, whereas the parameters of the flavin were retrieved from a previous study.[5] In all the simulations, the electrostatic interactions were computed using the particle mesh Ewald (PME) method using a cut-off of 12 Å. All the bonds involving hydrogen atoms were constrained using the SHAKE algorithm.

Minimization and MD simulations. The temperature and pressure were controlled using the Langevin thermostat and the Monte Carlo barostat, respectively. An integration time step of 2 fs was employed for all the simulations. The system was minimized through 10 000 steps of steepest descent followed by 5000 steps of conjugate gradient. A 500 ps NVT simulation was run from 0 to 300 K. Positional restraints were applied on heavy atoms during the heating using a force constant of 3 kcal/mol/Å^2. An additional NPT simulation of 1 ns was then run to allow the box to equilibrate at 300 K. NPT production simulations were performed for 2.5 μs for three independent replicas.

S2 Details on QM/MM excited-state calculations

In order to assess the TD-DFT level of theory employed in our excited-state calculations, we performed both TD-DFT and post-SCF ADC(2) and CC2 calculations in a QM/MM electrostatic embedding framework. Switching from QM/MM was necessary to ensure a proper comparison between CC2/ADC(2) and TD-DFT methods, as QM/AMOEBA is not implemented in TURBOMOLE. Furthermore, the increased computational cost of post-SCF methods forced us to use a

reduced QM region, containing only the isoalloxazine ring, for these calculations.

In all QM/MM calculations, the environment (protein, water, ribityl tail, and ions) was represented as point charges. MM charges were taken from the same force field used for the MD simulations. The ADC(2) and CC2 calculations were performed applying the RI approximation, as implemented in TURBOMOLE,[6] selecting the def2-SVP, def2-SVPD, def2-TZVP and TZVP basis sets. These calculations were performed on 110 structures extracted from dark-AppA and optimized as detailed in the Methods section.

S3 Supplementary Figures

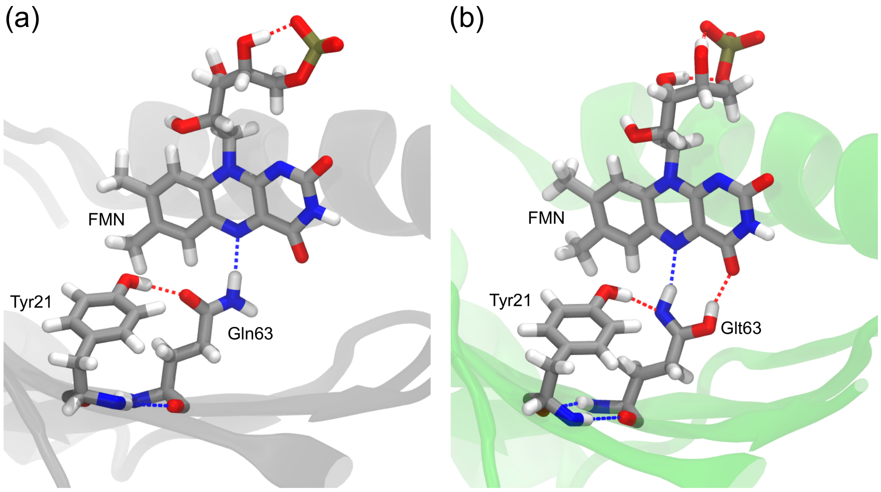

Figure S1: Hydrogen bond interactions between the flavin chromophore, Tyr21 and Gln63 in the binding pocket in (a) dark-AppA and (b) light-Appa classical MDs.

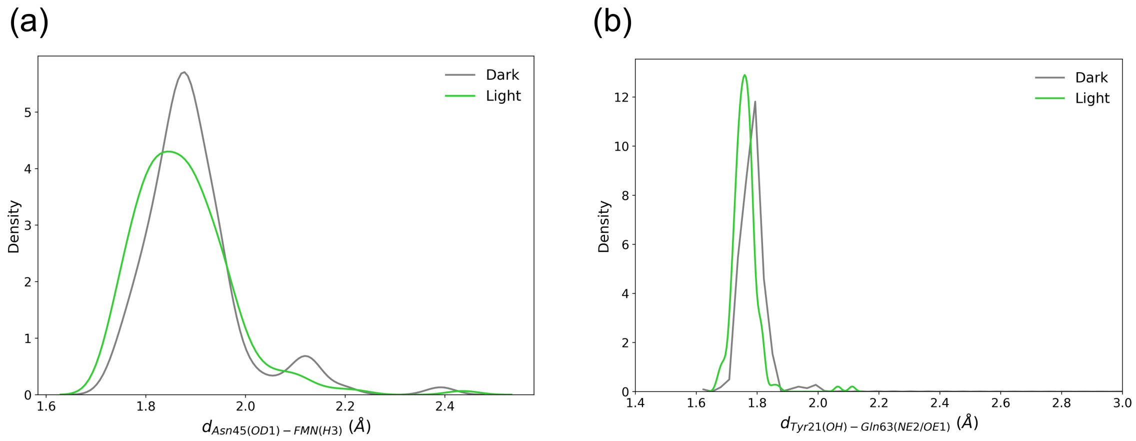

Figure S2: Distribution of the distance between (a) Asn45(OD1) and FMN(H3) (b) Tyr21(OH) and Gln63^{t}(NE2)/Gln63(OE1) for the optimized structures of dark-AppA and light-AppA.

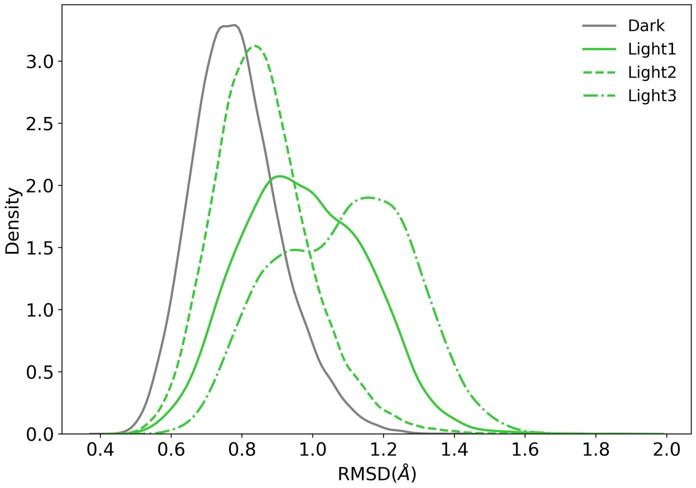

Figure S3: Distribution of the backbone RMSD values of the first 70 residues of the protein, calculated from the dark crystal structure (2IYG).

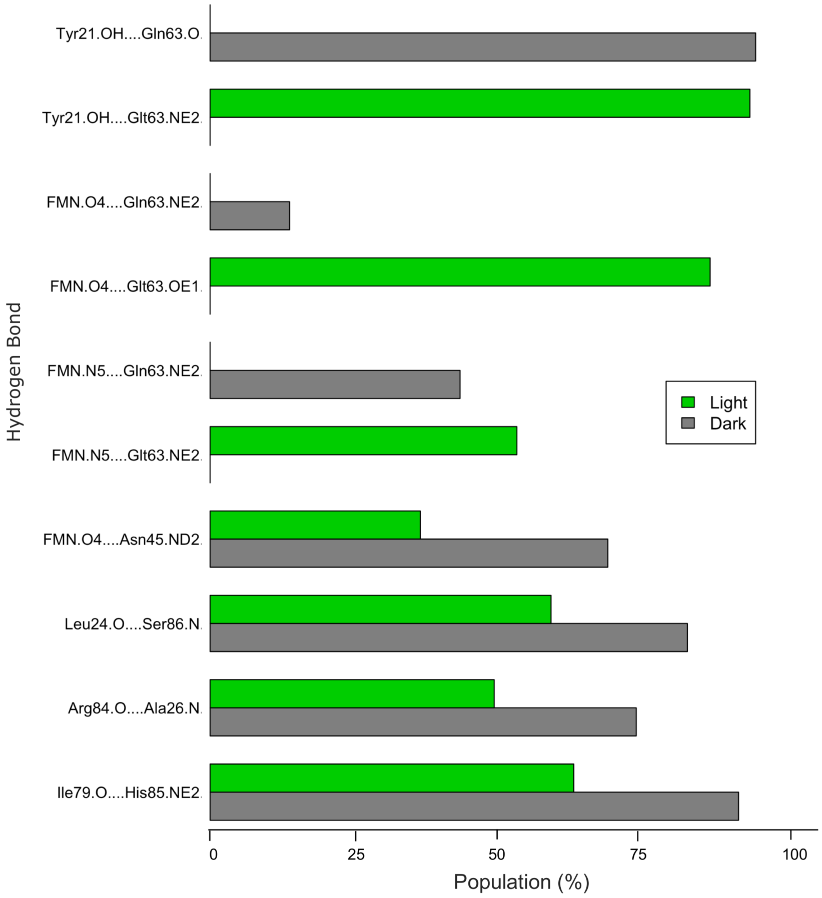

Figure S4: Bar plot showing the occurrence of hydrogen bonds within the binding pocket in dark-AppA (grey) and light-AppA (green) MDs.

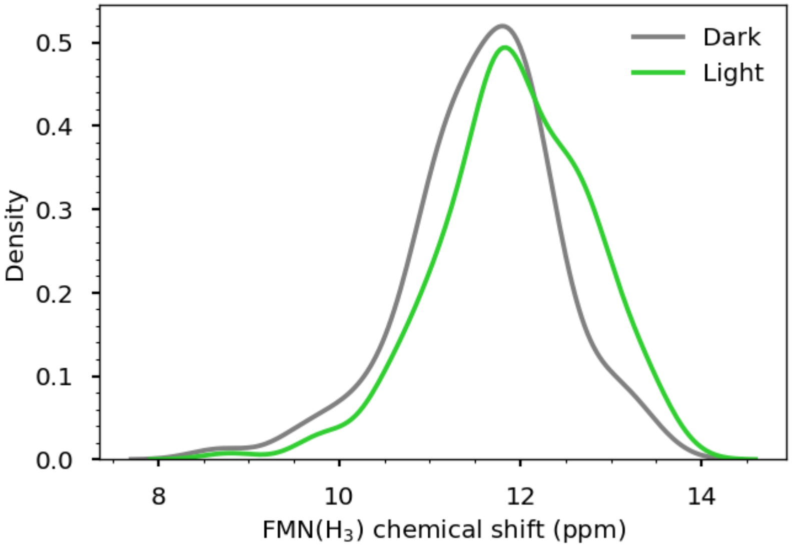

Figure S5: Distribution of chemical shifts for the H_3 atom of FMN in light-AppA and dark-AppA.

S4 Supplementary Tables

Table S1: Excitation energies ΔE and oscillator strengths for the first five singlets of FMN in dark-AppA calculated on the crystal structure[7] at the ωB97XD/6-31+G(d) level. The structure was optimized as detailed in the Methods section. The last column reports the excitation energies shifted by -0.35 **eV.**

	ΔE (eV)	Osc. strength	Shifted ΔE (eV)
S_1	3.103	**0.34**	2.753
S_2	3.731	0.008	3.381
S_3	4.127	**0.21**	3.777
S_4	4.453	0.0004	4.103
S_5	4.871	**0.34**	4.521

Table S2: Comparison between the vertical excitation energies (eV) for the bright states of AppA computed using different levels of theory and different basis sets. All calculations were performed with electrostatic embedding QM/MM on N =110 structures of Dark-AppA.

Method/Basis set	S_1	$S_{2'}$	$S_{2'}-S_1$
ADC(2) / def2-SVP	2.91	4.03	1.12
ADC(2) / def2-SVPD	2.80	3.84	1.05
ADC(2) / def2-TZVP	2.77	3.79	1.02
ADC(2) / TZVP	2.82	3.89	1.07
CC2 / def2-SVP	3.05	4.12	1.07
CC2 / def2-SVPD	2.93	3.92	0.99
CC2 / def2-TZVP	2.91	3.89	0.98
CC2 / TZVP	2.97	3.99	1.02
B3LYP / 6-31G(d)	2.94	3.67	0.73
B3LYP / 6-31+G(d)	2.89	3.59	0.70
B3LYP / 6-311+G(2d,p)	2.86	3.57	0.70
B3LYP / aug-cc-pVDZ	2.86	3.57	0.71
B3LYP / cc-pVDZ	2.94	3.69	0.76
ωB97XD / 6-311+G(2d,p)	3.20	4.00	0.80
ωB97XD / 6-31G(d)	3.29	4.12	0.84
ωB97XD / 6-31+G(d)	3.24	4.03	0.79
ωB97XD / aug-cc-pVDZ	3.20	4.01	0.81
ωB97XD / cc-pVDZ	3.28	4.14	0.86

References

(1) Case, D. A.; Ben-Shalom, I. Y.; Brozell, S. R.; Cerutti, D. S.; Cheatham, T. E.; III,; Cruzeiro, V. W. D.; Darden, T. A.; Duke, R.; Ghoreishi, D.; Gilson, M. K.; Gohlke, H.; Goetz, A. W.; Greene, D.; Harris, R.; Homeyer, N.; Izadi, S.; Kovalenko, A.; Kurtzman, T.; Lee, T. S.; LeGrand, S.; Li, P.; Lin, C.; Liu, J.; Luchko, T.; Luo, R.; Mermelstein, D. J.; Merz, K. M.; Miao, Y.; Monard, G.; Nguyen, C.; Nguyen, H.; Omelyan, I.; Onufriev, A.; Pan, F.; Qi, R.; Roe, D. R.; Roitberg, A.; Sagui, C.; Schott-Verdugo, S.; Shen, J.; Simmerling, C. L.; Smith, J.; Salomon-Ferrer, R.; Swails, J.; Walker, R. C.; Wang, J.; Wei, H.; Wolf, R. M.; Wu, X.; Xiao, L.; York, D. M.; Kollman, P. A. AMBER 2018. 2018; University of California, San Francisco.

(2) Lee, T.-S.; Cerutti, D. S.; Mermelstein, D.; Lin, C.; LeGrand, S.; Giese, T. J.; Roitberg, A.; Case, D. A.; Walker, R. C.; York, D. M. GPU-Accelerated Molecular Dynamics and Free Energy Methods in Amber18: Performance Enhancements and New Features. *J. Chem. Inf. Model.* **2018**, *58*, 2043–2050.

(3) Hashem, S.; Macaluso, V.; Nottoli, M.; Lipparini, F.; Cupellini, L.; Mennucci, B. From Crystallographic Data to the Solution Structure of Photoreceptors: the Case of the AppA BLUF Domain. *Chem. Sci.* **2021**, *12*, 13331–13342.

(4) Maier, J. A.; Martinez, C.; Kasavajhala, K.; Wickstrom, L.; Hauser, K. E.; Simmerling, C. ff14SB: Improving the Accuracy of Protein Side Chain and Backbone Parameters from ff99SB. *J. Chem. Theory Comput.* **2015**, *11*, 3696–3713.

(5) Schneider, C.; Sühnel, J. A Molecular Dynamics Simulation of the Flavin Mononucleotide-RNA Aptamer Complex. *Biopolymers* **1999**, *50*, 287–302.

(6) TURBOMOLE V7.2 2017, a development of University of Karlsruhe and Forschungszentrum Karlsruhe GmbH, 1989-2007, TURBOMOLE GmbH, since 2007; available from `http://www.turbomole.com` (accessed 10 May 2023).

(7) Jung, A.; Reinstein, J.; Domratcheva, T.; Shoeman, R. L.; Schlichting, I. Crystal Structures of the AppA BLUF Domain Photoreceptor Provide Insights Into Blue Light-mediated Signal Transduction. *J. Mol. Biol.* **2006**, *362*, 717–732.
